# Reluctance against the machine: Retrieval of observational stimulus–response episodes in online settings emerges when interacting with a human, but not with a computer partner

**DOI:** 10.3758/s13423-022-02058-4

**Published:** 2022-01-21

**Authors:** Carina G. Giesen, Klaus Rothermund

**Affiliations:** Department of Psychology, General Psychology II, Friedrich Schiller University Jena, Am Steiger 3, Haus 1, 07743 Jena, Germany

**Keywords:** Observational stimulus–response bindings, Event files, Observational learning, Episodic retrieval, Online interactions

## Abstract

Observing how another person responds to a stimulus creates stimulus–response (SR) episodes. These can be retrieved from memory on later occasions, which means that observed responses are utilized for regulating one’s own actions. Until now, evidence for storage and retrieval of observationally acquired SR episodes was limited to dyadic face-to-face interactions between two partners who respond in an alternating fashion. In two preregistered studies (total *N* = 252), we demonstrate for the first time that observational SR episodes can also be acquired in online interactions: Robust retrieval effects emerged when observers believe to be interacting with another person. In turn, retrieval effects were absent when observers believe to be interacting with a computer. Our findings show that feature-based binding and retrieval principles are pervasive and also apply to social interactions, even under purely virtual conditions. We discuss implications of our findings for different explanatory accounts of social modulations of automatic imitation.

**Supplementary Information:**

The online version contains supplementary material available at 10.3758/s13423-022-02058-4.

Recent findings indicate that merely observing a response from another person to a particular stimulus can result in an *observationally acquired stimulus–response* (SR) episode (synonymous terms are *bindings* or *event files*; Hommel 1998) in observers (Giesen et al., 2014; Giesen et al., 2016; Giesen et al., 2018; Giesen et al., 2021). Reencountering the same stimulus on subsequent occasions will retrieve the observationally acquired SR episode from memory, which impacts on observers’ performance, depending on whether the retrieved response is compatible with the appropriate response (producing facilitation) or incompatible (producing interference; statistically, retrieval of observationally acquired SR episodes is therefore reflected in an interaction of Stimulus Relation × Response Compatibility).

Observationally acquired SR episodes bear a close structural resemblance to social learning from observation (Bandura, 1986). Similar to social learning, retrieval of observationally acquired SR episodes is strongly affected by the social relevance between models and observers and is contingent on (a) situationally or chronically interdependent relations (e.g., cooperation/competition, Giesen et al., 2014, or interacting with one’s romantic partner, Giesen et al., 2018) and (b) positive vicarious feedback (Giesen et al., 2016). This insight is particularly important, as it suggests that basic processes like stimulus–response binding and retrieval are pervasive principles of action regulation (Frings et al., 2020; Henson et al., 2014), which are not limited to self-performed actions but also apply to social phenomena (Hommel, 2018; Hommel & Colzato, 2015; Hommel & Stevenson, 2021; Kim & Hommel, 2015, 2019; Ma et al., 2019).

So far, evidence for a modulation of retrieval of observationally acquired SR episodes by social relevance is limited to dyadic interactions between two interaction partners who respond in alternating fashion. Giesen and Frings (2021) studied observationally acquired SR episodes for videotaped responses that were observed on-screen. Surprisingly, in their study retrieval effects were unaffected by manipulations of visual perspective or group membership. For instance, retrieval effects emerged when videos had a perspective that was similar to observers’ views on their own hands (first-person perspective), but also when perspective was different from observers’ views (third-person perspective). Likewise, retrieval effects emerged when videos depicted a hand model with the same social group membership as observers (in-group model) but also when videos depicted a hand model from a different social group as observers (out-group model). Thus, retrieval effects of equal strength emerged for responses of high *and* low social relevance. This is at odds not only with the findings on retrieval of observationally acquired SR episodes in the dyadic face-to-face paradigm variant, but also with findings from other tasks which measure compatibility effects in nondyadic situations as a consequence of mentally representing observed actions (see Table 1 for an overview and description of different experimental approaches to measure imitative tendencies). Social modulations of observing a motion sequence on-screen depicting an irrelevant yet (in-)compatible action are documented for the automatic imitation task (Brass et al., 2001; Butler et al., 2016; Cracco, Genschow, et al., 2018b; for an overview, see Cracco et al., 2018a, b). For instance, individuals imitate others less strongly when they observe actions from a third-person perspective compared with a first-person perspective (Bortoletto et al., 2013; Genschow et al., 2013; Lamm et al., 2007; Vogt et al., 2003) or when they face out-group compared with in-group members (Genschow & Schindler, 2016; but see Genschow, Westfal, et al., 2021b, for a failed replication of this finding). Similarly, joint Simon effects emerge for situations in which participants believe to observe the action of a human interaction partner seated in an adjacent cubicle (Tsai et al., 2008).

**Table 1:** Overview of different experimental approaches to study imitative behaviour

Description	Dependent measure	Characteristics
Mimicry studies (e.g., Chartrand & Bargh, 1999): During an interaction, unintentional copying of manners, gestures, postures, and other motor behaviours of the partner are investigated.	Frequency of a specific behaviour (e.g., face touching, foot shaking) depending on the behaviour of the model.	Mimicked behaviour is irrelevant for action regulation, but relevant for social interactions. Mimicry and automatic imitation are often considered to measure similar processes (Wang & Hamilton, 2012), but this claim can be debated (Genschow et al., 2017).
Automatic imitation task (Brass et al., 2001): Participants have to lift their index finger when the number “1” is presented and lift their middle finger if the number “2” is presented. Participants simultaneously observe an index-finger or middle-finger movement in a picture sequence on-screen, which is irrelevant for the task. Observed and to-be-performed actions are compatible or incompatible; a third, no-movement condition serves as neutral baseline.	Trial performance (RT, accuracy) as a function of compatibility between observed action and to-be-executed action	Simultaneously observed actions facilitate or interfere with task execution: Typically, movement execution in response to the numbers is faster and more accurate if compatible actions are observed but is impeded if incompatible actions are observed (compared with the baseline).
Joint Simon task (Sebanz et al., 2003): Pictures of a finger with a red or green ring are presented to two participants. In a color categorization task, one participant responds only to “red” stimuli, and the other participant responds only to “green” stimuli. The finger points either left or right (i.e., in the direction of either participant), which is irrelevant for the task. Pointing direction and ring color can be compatible (i.e., finger points towards the participant who has to respond), incompatible (finger points towards the participant who does not have to respond), or neutral (finger points towards the middle).	Trial performance (RT, accuracy) as a function of compatibility between to-be-executed action and irrelevant stimulus dimension (e.g., pointing direction).	Performance is better on compatible trials and is worse on incompatible trials, compared with the baseline. This joint compatibility effect is taken as an indicator that participants automatically co-represent the action of their co-actor, and hence face interference if the finger points toward the other participant although it is their turn to respond (but see Dittrich et al., 2012; Dolk et al., 2013, for an alternative explanation).
Observational stimulus–response binding (Giesen et al., 2014): A color categorization is shared between two participants. During prime trials, Person A classifies the color of a word; Person B observes the response to the same word, which is visible to them only in white. In the following probe trials, former prime observers have to classify the color of a word. Responses and words either repeat or change from prime to probe.	Probe trial performance (RT, accuracy) as a function of word relation (repetition vs. change) and compatibility between prime and probe responses.	Stimulus repetition (compared with stimulus change) leads to facilitation for response repetitions, but leads to interference for response changes. This effect pattern is taken as an indicator for incidental bindings between observed prime responses and prime stimuli. Stimulus repetition in the probe retrieves this binding, which facilitates or hampers performance, depending on whether the retrieved response is appropriate or not. Retrieval of observational SR episodes is contingent on social relevance of interaction partners.

We propose that the absence of social modulation of retrieval of observationally acquired SR episodes in the study by Giesen and Frings (2021) can be explained by subtle differences in the way stimuli and responses were displayed that promoted feature-based binding effects even in situations of low social relevance, which were absent in the dyadic face-to-face paradigm. In the face-to-face paradigm, people only see a word stimulus on-screen, whereas the response (pressing a red or green push button) is observed *outside* the screen and in the periphery; also, the stimulus disappears as soon as the interaction partner initiates the response. In the video-based variant, videos are presented in the lower part of the screen. This region is known to be perceived as visual foreground, and presentations in this region promote binding and retrieval (Frings & Rothermund, 2017). Also, stimuli and video-taped responses are grouped both spatially (forming a perceptual unit framed by the monitor) and temporally (stimulus and response disappear as soon as videos end), yet grouping is known to promote binding and retrieval, too (Frings & Rothermund, 2011). With this in mind, one could argue that figure–ground segmentation and Gestalt grouping alone are sufficient to produce reliable retrieval effects for observed stimulus–response combinations even in situations of low social relevance.

In the present study, we removed all of these differences (see the Method section for details) to investigate whether observationally acquired SR episodes are prone to a modulation by social relevance also in virtual interactions—that is, in an online task. In two experiments, half of the participants were made believe they were engaging in an interactive color classification task together with another person, whereas the other half of the participants were told they were interacting with a computer. Animacy belief is a robust and reliable social moderator on compatibility effects in the automatic imitation task (Gowen et al., 2016; Klapper et al., 2014; Liepelt et al., 2010; Liepelt & Brass, 2010; Press et al., 2006; Stanley et al., 2007) and in the joint Simon task (Müller et al., 2011; Tsai & Brass, 2007; Tsai et al., 2008), reflecting stronger effects when participants believe to be observing actions from a human partner versus a computer or robot. Hence, we expected to find retrieval of observationally acquired SR episodes for participants who believed to be interacting with a human partner; in turn, retrieval effects should be absent for participants who were told to be interacting with the computer. To anticipate, our initial reasoning was supported (Experiment 1). We then ran an exact replication with an even larger sample to assess the robustness of our findings (Experiment 2). Methods and results are presented together for both experiments.

## Method

### Ethics vote, preregistration, and open access

Ethical approval was granted for both experiments by the Ethics Committee of the FSU Jena (FSV 21/034). Prior to data collection, the exact method, design, hypotheses, data preparation, and planned analyses were preregistered online at the Open Science Framework (OSF; Experiment 1: https://osf.io/8ktwv; Experiment 2: https://osf.io/ptsx8). All stimulus materials, data, and analyses scripts will be made available after initial acceptance of the paper (link for review: https://osf.io/68uvx/?view_only=eb141ed62f5e445193a59bb855629d27).

### Required sample size and a priori power calculations

We ran a priori power calculations to estimate required sample sizes with 1 − ß = .80 and α = 0.05, for independent *t* tests (one-tailed) with G*Power 3.1 (Faul et al., 2007). For Experiment 1, no prior effect sizes were available, which is why we calculated the required sample size based on a medium-sized effect (*d* = 0.5). Accordingly, a total of *n* = 102 (51 per group) is needed to guarantee a sufficiently powered study. For Experiment 2, a priori power calculations were based on the size of the effect that was obtained in Experiment 1 (*d* = 0.39).^1^ To be able to detect an effect of this size with sufficient power (1 − ß = .80), a total of *n* = 164 participants (82 per group) are needed.

#### Participants

In total, 103 participants were recruited online at Prolific Academic (https://www.prolific.co/) for Experiment 1. Five participants had to be excluded due to excessive error rates (>25% errors in the memory test); four participants did not pass the practice block; one participant took part twice; hence, the second participation was excluded. Data of *n* = 93 participants were analyzed (33 females, 58 males, two gender not reported; *M*_age_ = 26.5 years). For Experiment 2, 161 new participants were recruited online at Prolific Academic. According to the same criteria as in Experiment 1,^2^ two participants were excluded because of excessive error rates or incomplete data. Data of *n* = 159 participants were analyzed (58 females, 97 males, four diverse, *M*_age_ = 25.0 years). All participants were prescreened to be Native German speaking, aged between 18 and 35 years, with a Prolific approval rate of at least 65%–100% in prior studies, using Windows 10 as an operating system and running the experiments on a notebook or desktop computer. Both experiments had a median duration of 22 minutes and participants received £2.75 (€3.19) for taking part. All participants gave informed consent via key press prior to taking part in the studies.

#### Design

Both experiments comprised a 2 (stimulus relation: word repetition vs. change) × 2 (response compatibility: compatible vs. incompatible) × 2 (interaction partner: human vs. computer) mixed-factors design. Probe reaction times (RT) served as dependent variable of interest.

#### Materials and procedure

Experiments were programmed with E-Prime 3 and were converted for online data collection with E-Prime Go 1.0. At the start of each experiment, demographic information (gender, age, handedness, native language) was collected, followed by the consent page. If participants consented to take part, instructions followed; otherwise, the study was terminated. Participants were informed that they would perform an interactive color classification task together with another person.

***Human vs. computer interaction conditions*** **.** Participants were then randomly assigned to either the *human partner* (Experiment 1: *n* = 47; Experiment 2: *n* = 68) or *computer partner* (Experiment 1: *n* = 46; Experiment 2: *n* = 91) condition (note that random assignment of participants to conditions produced unequal group sizes in Experiment 2). Participants in the human partner condition were supposedly connected with their interaction partner and were asked to write a short message to welcome their partner. When they finished their message, they were prompted with a message by their putative interaction partner, accompanied by name and age information. Participants had to wait occasionally for their partner to finish reading instructions or executing responses. All of this was done to induce the feeling that participants in the human partner condition interacted live with another actual person. In fact, all partner interactions and the messages were scripted, and participants in the human condition interacted with a computer program, too. Participants in the computer partner condition were informed that connecting was not possible as no online interaction partner was available when the study started. Thus, they would continue with the study with a computer program as their partner.

***Assessment of observational SR episodes and retrieval*** **.** To assess observationally acquired SR episodes, we used a sequential priming paradigm: Participants were instructed that they would perform an interactive color classification task in turns with their partner (depending on the condition, partner either referred to an alleged human interaction partner or the computer). They were informed that a word would appear centrally on-screen within a squared rectangle. When the word font was red or green, it was participants’ turn to respond and categorize the color and press A (left key) for red and L (right key) for green. Each key press lit up a *virtual* red or green response button, displayed in the upper left and right corner of the screen (i.e., the screen region which is perceived as visual background to counteract ad hoc binding; cf. Frings & Rothermund, 2017) and simultaneously elicited a clicking sound. When the word appeared in white, however, it was their partner’s turn to respond. Participants could observe their partner’s key press, as either the red or green virtual response button would light up on-screen together with a clicking sound in the same fashion as for their own responses. Participants were further told to pay close attention to their partner’s responses and memorize them, as they would be confronted with occasional memory test trials probing for the responses that were given by their partners. Participants completed a brief instruction check in which they had to answer two questions about the task. If they did not answer these with 100% accuracy, participants were redirected to the beginning of the instruction to reread them until they understood the task and passed the instruction check. After that, a brief practice block of 16 prime–probe sequences followed; the practice block was repeated if participants made more than 20% errors in the color classification task or more than 50% responses slower than 1,000 ms. Upon successful completion of the practice block, the main block started which comprised of 128 prime–probe sequences that were constructed as follows: The interaction partner always responded during the prime display; participants always responded during the probe display. Thus, participants observed responses to particular stimuli during the prime display and carried out probe responses that were compatible or incompatible with previously observed responses during the probe display. For 50% of all sequences, observed prime and executed probe responses were compatible (green–green; red–red); for the rest, they were incompatible (green–red; red–green). Orthogonally to response compatibility, the stimulus relation was manipulated: On 50% of all sequences, the same word was presented in prime and probe (word repetition); on the remaining sequences, two different word stimuli were presented in prime and probe (word change). Word stimuli were randomly sampled from 25 neutral, monosyllabic or disyllabic German adjectives. Probe color was counterbalanced (50% red; 50% green).

**Fig. 1:**
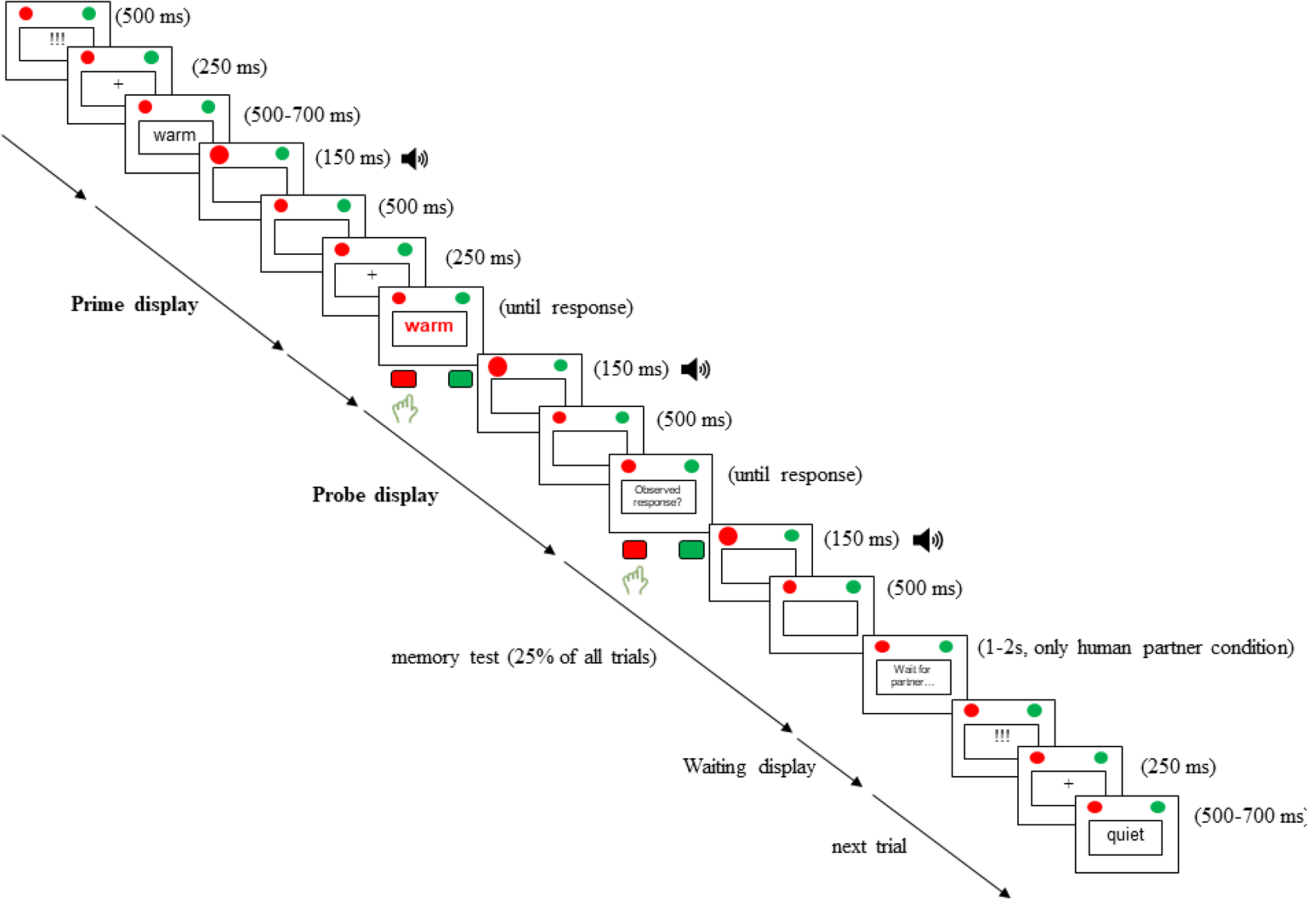
Example of prime–probe sequence. Stimuli are not drawn to scale. For illustrative purposes, foreground and background colors are inverted. Stimuli in boldface were presented in red/green; stimuli in normal face were presented in white

The prime–probe sequences were as follows (see Fig. 1): Each display showed a red and green virtual button in the upper left and right display corner. All stimuli were presented in the screen center, surrounded by a white square to visually separate stimuli and response buttons (Frings & Rothermund, 2011). Each trial sequence started with a ready signal (!!!) presented centrally (500 ms), followed by a fixation cross (250 ms). Then, the prime display started: A white word appeared centrally; after a variable interval of 500–700 ms, the word disappeared, and either the red or green response button lit up: This illusion was created by presenting a picture of a larger button for 150 ms, followed by the standard button for 500 ms. Simultaneously, a buzzer sound (duration: 300 ms) was played. Accidental prime responses by the participant elicited feedback (“wrong person,” 1,000 ms). Another fixation cross followed (250 ms), after which the probe display started: A red or green word appeared centrally (until response). Depending on whether the red (A) or green (L) key was pressed, either the red or green button lit up and elicited the buzzer sound; timing was identical to prime displays. Erroneous probe responses elicited feedback (“wrong key,” 1,000 ms). After 32 randomly chosen probe displays (25% of all probes), a memory test followed. Participants were asked to press the response key that corresponded to the observed response (duration until response). Depending on whether the red (A) or green (L) key was pressed in response to the memory prompt, either the red or green button lit up and elicited the buzzer sound. Erroneous memory test response elicited feedback (“inaccurate observation,” 1,000 ms). For participants in the human partner condition, after another randomly chosen probe display, a waiting screen appeared with the prompt “Waiting for partner to respond” (variable duration of 1000, 1500, 1750, or 2000 ms) to convey the impression that the alleged interaction partner performed a memory test. The trial sequence ended with a blank screen (250 ms).

After a block of 32 prime–probe sequences, participants received interim feedback on the past block (% errors in color categorization, % slow responses, % memory errors) based on their own performance. Participants in the human partner condition also received feedback regarding the partner’s performance; however, this was again scripted.

When the task was completed, a couple of questions were asked on-screen. First, participants were asked to write down what they thought the study was about. Second, participants were asked to indicate with whom they interacted (options: computer, human, no idea) by selecting one of three buttons on the screen via mouse click. Then, participants in the human partner condition were asked to remember the name and age of their interaction partner (this functioned to check whether participants remembered details about their partner). Last, all participants were asked to rate how realistic they experienced the interaction via mouse click on a 9-point Likert scale (1= *very unrealistic*; 5= *neutral*; 9 = *very realistic*). When the questions were completed, all participants received completion codes for participation and were fully debriefed.

### Data preparation

Prior to analyzes, probe responses were discarded either because of color classification errors (Experiment 1: 1.5%; Experiment 2: 1.4%) or because of errors in the memory test (Experiment 1: 4.5%, overall: 1.1%; Experiment 2: 4.1%, overall 1.0%). Also, probe responses faster than 200 ms or slower than 1.5 interquartile ranges above the 75th percentile of the individual RT distribution were regarded as RT outliers (Tukey, 1977) and were excluded (Experiment 1: 3.7%; Experiment 2: 3.7%). Mean probe RT for the factorial design are presented in Table 2. For each experiment, we computed effect scores for retrieval of observationally acquired SR episodes for each participant that reflected the Stimulus Relation × Response Compatibility interaction (see Table 2 for computation). Positive values on this score reflect a pattern that indicates retrieval of observational SR episodes (i.e., performance benefits due to stimulus-based retrieval of compatible observed responses and performance costs due to stimulus-based retrieval of incompatible observed responses).

**Table 2:** Probe performance *M* (*SD*) and control variables in the observational SR binding paradigm

			Human partner		Computer				
			C	IC	C	IC			
Experiment 1		Stimulus repetition (SR)	484 (67)	495 (80)	469 (68)	469 (71)			
		Stimulus change (SC)	493 (77)	487 (72)	475 (70)	472 (69)			
		ΔSC-SR	9* [3.9]	−8* [3.5]	6 [3.3]	3 [3.5]			
		S × R interaction scores	17** [5.7]		3 [4.1]				
Experiment 2		Stimulus repetition (SR)	476 (65)	481 (75)	468 (66)	470 (62)			
		Stimulus change (SC)	484 (65)	475 (65)	474 (65)	476 (67)			
		ΔSC-SR	8**[2.6]	−6*[2.8]	6**[2.1]	6*[2.3]			
		S × R interaction scores	14*** [4.1]		0 [2.9]				
**Memory test performance (error rate)**			*M*		*M*		*t*	*df*	*p*
		Experiment 1	5.9		4.5		1.07	91	.289
		Experiment 2	3.6		4.6		1.21	157	.228
**Postexperimental questions**			**Human partner**		**Computer**		*t*	*df*	*p*
Experiment 1	Name / Age remembered correctly		100% / 79%		–				
	Whom did you interact with?	Computer	72%		100%				
		Human	26%		0%				
		No idea	2%		0%				
	How realistic did you perceive the interaction?		4.3		5.9		3.99	91	<.001
Experiment 2	Name / Age remembered correctly		97% / 85%		–				
	Whom did you interact with?	Computer	74%		99%				
		Human	21%		1%				
		No idea	5%		0%				
	How realistic did you perceive the interaction?		4.4		6.1		5.13	157	<.001

C = compatible probe response. IC = incompatible probe response. Standard error of the mean in brackets. S × R interaction score = (ΔSC-SR)_C_ − (ΔSC-SR)_IC._ *p < .05. ** *p* < .01. ***p ≤ .001. Asterisks denote that effects are significantly different from zero

## Results

### Retrieval of observational SR episodes

**Fig. 2:**
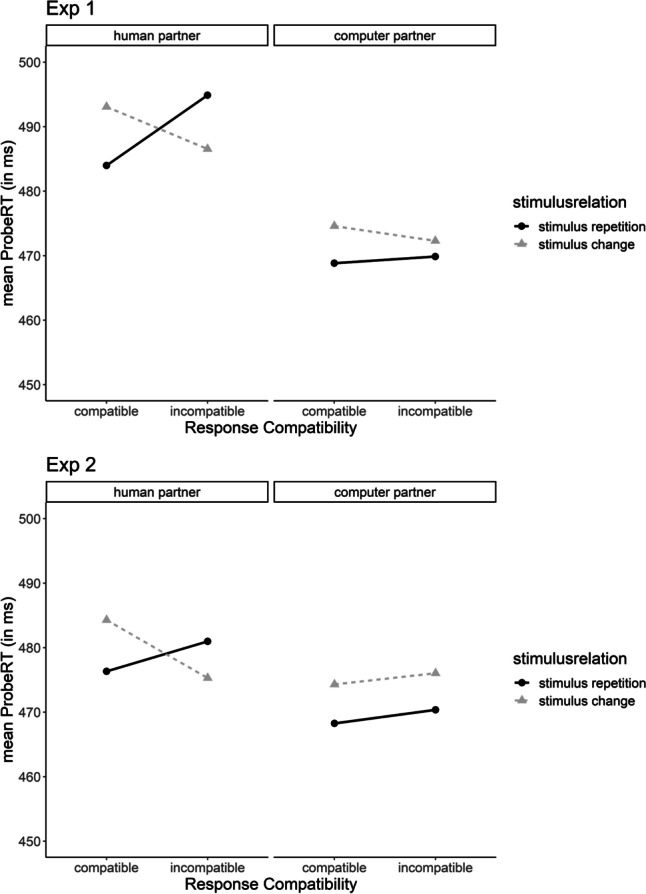
Probe performance (RT, in ms) in Experiment 1 (top) and Experiment 2 (bottom) as a function of stimulus relation, response compatibility, and interaction partner condition. As can be seen, a disordinal interaction between the factors stimulus relation and response compatibility is always present for participants who believed to be interacting with a human partner (left side), which is indicative of retrieval of observationally acquired SR episodes: Stimulus repetition (compared with stimulus change) produced performance benefits when to-be-executed probe responses were compatible with observed prime responses, but produced performance costs when to-be-executed probe responses were incompatible with observed prime responses. In turn, the interaction is absent for participants who were told to be interacting with a computer partner (right side)

To test our directional hypothesis, observational SR binding and retrieval effect scores were analyzed as a function of interaction partner condition in one-tailed, independent-samples *t* tests.^3^ This difference was significant both in Experiment 1, *t*(91) = 2.01, *p* = .024, *d* = 0.42, and Experiment 2, *t*(157) = 2.72, *p* = .004, *d* = 0.43, indicating that effect scores were significantly larger for participants in the human partner condition (Experiment 1: S×R_human_ = 16 ms; Experiment 2: S×R_human_ = 14 ms) than in the computer partner condition (Experiment 1: S×R_computer_ = 2 ms; Experiment 2: S×R_computer_ = 0 ms; see Table 2, Fig. 2). Follow-up tests showed that observational SR binding and retrieval effect scores differed significantly from zero for the human partner condition in Experiment 1, *t*(46) = 2.83, *p* = .003 (one-tailed), *d*_*z*_ = 0.41, and Experiment 2, *t*(67) = 3.33, *p* = .001 (one-tailed), *d*_*z*_ = 0.40. This was not the case for the computer partner condition, neither in Experiment 1, *t*(45) = 0.69, *p* = .493, *d*_*z*_ = 0.10, nor in Experiment 2, *t*(90) = 0.13, *p* = .899, *d*_*z*_ = 0.01, meaning that no evidence for observational SR binding and retrieval was obtained for this condition (Fig. 2).

### Control variables

#### Memory test performance

Performance in the memory test was compared as a function of interaction partner to assess whether the difference in retrieval of observational SR episodes might be due to the fact that participants in the computer partner condition paid less attention to observed responses, which would result in weaker effects. This was not the case, as memory performance (indicated by error rates) did not differ between interaction partner conditions, neither in Experiment 1, nor in Experiment 2 (cf. Table 2).

#### Postexperimental questions

Nearly all participants in the human partner condition correctly remembered the name of their alleged interaction partner in both studies; the majority also remembered the correct age (Table 2). Interestingly, whereas all participants of the computer partner condition reported that they interacted with a computer in both experiments, only some participants of the human partner condition reported that they interacted with another person (Table 2), and most reported that they interacted with a computer. Also, participants in the human partner condition perceived the interaction as significantly less realistic than participants in the computer partner condition in both experiments (Table 2). These findings most likely reflect a demand effect, as the questions probably made participants second-guess the nature of the study. We come back to this issue in the General Discussion (see also Supplementary Material).

## General discussion

The present findings are clear-cut: In two preregistered experiments, we obtained robust evidence for retrieval of observationally acquired SR episodes in an online setup for participants who believed to be interacting with another person. In turn, retrieval effects were virtually absent for participants who were told to be interacting with the computer. This is the first demonstration that retrieval of observationally acquired SR episodes in online settings is prone to the influence of social modulatory effects.

Before addressing theoretical implications, we want to discuss alternative explanations for the present findings. First, one could argue that participants in the computer partner condition simply paid less attention to observed responses, which would result in weaker or even completely absent binding and retrieval effects. If that were the case, memory test performance should be poorer for participants in the computer partner condition. However, error rates did not differ between both interaction partner conditions. Second, in the postexperimental questionnaire, a lot of participants from the human partner condition actually reported that they interacted with a computer. However, we believe that this is a demand effect that most likely reflects a postexperimental adjustment rather than true insight into the manipulation. This is based on two reasons: (a) If participants really second-guessed the human interaction partner condition and actually believed to be interacting with the computer, retrieval effects should have been absent as was the case for participants who were informed to be interacting with the computer right from the start. This was clearly not the case, as we obtained robust retrieval effects in the human partner condition. (b) Nevertheless, we ran an additional analysis (see Supplementary Material) only for participants in the human partner condition to assess whether retrieval effects were reduced or absent for those participants who reported to be interacting with the computer in the postexperimental questionnaire. Importantly, retrieval effects did not differ statistically as a function of reported interaction partner; if anything, the data pattern showed a trend in the reverse direction (i.e., stronger retrieval effects for participants of the human partner condition who later reported to have interacted with the computer). This data pattern argues against the possibility that these participants second-guessed the nature of the manipulation during the study. Hence, we believe it more likely that asking participants after their interaction partner brought them to change their opinion postexperimentally for the sake of appearance, thereby producing demand effects. Third, we interspersed occasional waiting displays that followed memory tests in the human partner condition. This was done to convey the impression that interaction partners were not yet finished with reporting remembered responses. However, one could argue that waiting displays had the unintentional effect of rendering the upcoming prime–probe sequence more distinct in memory, due to a longer time interval in between the current and subsequent prime–probe sequence. If some prime displays were more distinct, this would be beneficial for retrieval, as the memory episode is easier to discriminate from temporally closer episodes. This could explain why retrieval effects were selectively stronger in the human partner condition. To rule out this alternative explanation, we conducted another post hoc analysis (see Supplementary Material) in which we coded presence vs. absence of memory test in the preceding prime–probe sequence as a factor. However, this analysis showed that this factor did not modulate the size of retrieval effects. Hence, we can also discard this alternative explanation.

### Theoretical implications

Our data bear a number of theoretical implications. First, they support the view that the finding of unconditionally strong retrieval effects of observationally acquired SR episodes that were reported in Giesen and Frings (2021) are an artifact, which is unrelated to social information processing resulting from ad hoc feature binding due to perceptual grouping and figure–ground segmentation. These conditions produced reliable retrieval effects by default and independently of social contexts, that is, even in situations of low social relevance.

Second, our data converge with previous findings from related paradigms that investigated imitative or joint compatibility phenomena as a consequence of mentally representing observed actions in showing that animacy belief is a strong social modulatory factor, reflecting stronger compatibility effects when participants believe to be observing actions from a human versus computer or robot partner (see Cracco et al., 2018a, b, for a meta-analysis of the effects of social modulations in the imitative action paradigm). Our data represent first-hand evidence that this modulation also applies to observationally acquired SR episodes and retrieval thereof, which means that people utilize observed responses for regulating their own actions.

Third, our findings can be related to current theories^4^ on social modulations of compatibility-based measures of imitative behaviors. For instance, some authors argue that people use imitation either consciously (Wang & Hamilton, 2012) or unconsciously (Chartrand & Bargh, 1999; Chartrand & Dalton, 2009) as a tool to satisfy motives of social affiliation. According to these motivational accounts, participants should imitate more strongly when they have the goal to affiliate with others (Lakin & Chartrand, 2003). Other theoretical approaches are based on ideomotor principles and associative learning (Brass & Heyes, 2005; Greenwald, 1970; Heyes, 2010; Prinz, 1990). Accordingly, actions are produced by anticipating their sensory effects. As a consequence of this learnt association, observing an action (and its sensory effects) will mentally activate corresponding motor codes in the observer, which implies that people mentally represent their own as well as the other persons’ actions in terms of feature codes (Hommel, 2018). The activated motor code can then be used for imitating the model’s action. In this regard, imitative tendencies represent learnt responses that evolved as a consequence of self-observation and social interaction with other individuals (e.g., as a result of being imitated; Cook et al., 2014; Efferson et al., 2008; Ray & Heyes, 2011). As self-other overlap is a function of perceived similarity (Hommel & Colzato, 2015), individuals who are perceived as more similar to oneself should be imitated more strongly (Genschow, Cracco, et al., 2021a). Our findings are consistent with both theoretical accounts: On the one hand, people may have felt a stronger affiliation goal when interacting with human compared with nonhuman partners. On the other hand, it is reasonable to assume that participants in the human partner condition perceived their interaction partner as more similar to themselves, which obviously was not the case when believing to interact with the computer.

It is particularly noteworthy that findings from the observational SR binding paradigm bear a close structural resemblance to findings on observational learning, which provokes the idea to unite binding and retrieval principles with social learning theory. In this respect, social learning theory might provide a more parsimonious and integrative approach to explain imitative behaviors, as it can easily integrate existing theoretical approaches to explain social modulations of imitative response tendencies. According to Bandura (1986); see also Ahn et al., 2020), people do not copy any observed action per se. Instead, four constituent processes are crucial to obtain imitative behaviors and observational learning: (I) Models have to attract observers’ interest and appear worthy of imitation. This holds true for models that are perceived as personally relevant, similar, or competent. (II) Observed actions have to be encoded in memory in the form of symbolic representations—from today’s perspective, one may assume that what Bandura had in mind is conceptually similar to *common coding* (Prinz, 1990). (III) Observers then have to rely on these symbolic representations to guide their own performance. (IV) Perceived consequence of copying the model will strongly influence whether or not observed actions will be imitated by observers. If observed behaviors were vicariously reinforced or fulfill deprived motives, imitation is more likely. Thus, social learning theory integrates elements of self-overlap theories that apply ideomotor and associative learning principles to the social realm (Processes I–III) as well as motivational accounts (Process IV) and thus might serve as an integrative theory that can be applied to explain current research findings on imitative behaviors. Yet more research is needed to systematically test and substantiate this reasoning.

Fourth, the present data fit well with existing evidence of social relevance modulations that was gathered in the dyadic face-to-face version of the observational SR binding paradigm (Giesen et al., 2014; Giesen et al., 2016; Giesen et al., 2018; Giesen et al., 2021). On a more general level, our data document that basic binding and retrieval principles are not limited to self-performed actions, but are vividly active also in the social realm (for similar conclusions, see Hommel, 2018; Hommel & Colzato, 2015; Hommel & Stevenson, 2021; Kim & Hommel, 2015). Importantly, our findings demonstrate that social learning from observation is not limited to live, face-to-face interactions, but also occurs in virtual, online interactions. From a practical perspective, these findings attest to the fundamental relevance that televised and digital media have for the acquisition of new behavior. From a more methodological perspective, our paradigm provides researchers with an elegant tool to further study the modulation of basic processes of observational SR binding and retrieval by social, emotional, and motivational factors.

## Supplementary Information

ESM 1 (DOCX 639 kb)

## Footnotes

1 When rerunning the analysis, we noticed a small error in the R script for Experiment 1 that excluded too many trials from probe RT. Correcting this error produced a slightly higher effect size of *d* = 0.42 (see Results section for details). For reasons of consistency, we report the effect size estimate of *d* = 0.39 that was used in sample size calculation in the preregistration of Study 2.

2 Note that the preregistration of Experiment 2 had a typo and reports a cutoff of 20% for errors in the memory test. Since Experiment 2 was planned as an exact replication of Experiment 1, we analysed the data of both experiments with the same error cutoff threshold of 25% max.

3 We want to clarify that the independent *t*-test on interaction effect scores between both interaction partner conditions is mathematically equivalent to the test of the three-way interaction in a 2 (stimulus relation: repetition vs. change) × 2 (response compatibility: compatible vs. incompatible) × 2 (interaction partner: human vs. computer) mixed-factors analysis of variance (ANOVA), with *t*^2^ = *F* and *df* = 1 that was specified in the preregistration. Given that the ANOVA only yields *F* values, which cannot be submitted to a one-tailed test to falsify a directional hypothesis, we decided to report *t* tests in the main analyses. Note that the directional hypothesis is explicitly mentioned in both preregistrations. We provide full ANOVA results for both experiments and for a joint analysis in the Supplementary Material.

4 We thank an anonymous reviewer for making us aware of these connections.
