# Supplementary material for: Reluctance against the machine: Retrieval of observational stimulus–response episodes in online settings emerges when interacting with a human, but not with a computer partner
Source: Psychon Bull Rev. 2022 Jan 21;29(3):855–65. doi: 10.3758/s13423-022-02058-4 (PMC9166856; doi:10.3758/s13423-022-02058-4)
Supplement: Supplementary file 1 — (DOCX 639 kb) [file 13423_2022_2058_MOESM1_ESM.docx]

**Supplementary Material**

**Supplementary Analysis I: ANOVA results for Experiments 1 and 2**

| **Supplement Table 1** | |  |  |  |  |  |
| --- | --- | --- | --- | --- | --- | --- |
| Separate ANOVA results for probe performance in Experiment 1 and 2. | | | | | | |
|  | Variables | *df 1* | *df 2* | *F* | *p* | *η*_p_² |
| Experiment 1 |  |  |  |  |  |  |
|  | Interaction Partner (P) | 1 | 91 | 1.58 | .212 | .02 |
|  | Stimulus Relation (S) | 1 | 91 | 1.55 | .217 | .02 |
|  | Response Compatibility (R) | 1 | 91 | 0.11 | .741 | .00 |
|  | S × P | 1 | 91 | 1.09 | .299 | .01 |
|  | R × P | 1 | 91 | 0.35 | .558 | .00 |
|  | S × R | 1 | 91 | 8.85** | .004 | .09 |
|  | S × R × P | 1 | 91 | 4.03* | .048 | .04 |
| Experiment 2 |  |  |  |  |  |  |
|  | P | 1 | 157 | 0.46 | .500 | .00 |
|  | S | 1 | 157 | 10.20** | .002 | .06 |
|  | R | 1 | 157 | 0.01 | .927 | .00 |
|  | S × P | 1 | 157 | 3.75 | .054 | .02 |
|  | R × P | 1 | 157 | 1.12 | .292 | .01 |
|  | S × R | 1 | 157 | 6.26* | .013 | .04 |
|  | S × R × P | 1 | 157 | 7.38** | .007 | .04 |
| *Note.* *df*= degrees of freedom. **p*<.05. ***p*<.01. ****p*<.001. | | | | | | |

**Supplementary Analysis II: ANOVA results for joint analysis**

| **Supplement Table 2** | |  |  |  |  |  |
| --- | --- | --- | --- | --- | --- | --- |
| ANOVA results for joint analysis of Experiment 1 and 2. | | | | | | |
|  | Variables | *df 1* | *df 2* | *F* | *p* | *η*_p_² |
|  | Experiment (E) | 1 | 248 | 1.76 | .186 | .01 |
|  | Interaction Partner (P) | 1 | 248 | 0.27 | .605 | .00 |
|  | Stimulus Relation (S) | 1 | 248 | 10.43** | .001 | .04 |
|  | Response Compatibility (R) | 1 | 248 | 0.07 | .788 | .00 |
|  | P × E | 1 | 248 | 0.42 | .519 | .00 |
|  | P × S | 1 | 248 | 4.61* | .033 | .02 |
|  | E × S | 1 | 248 | 0.38 | .538 | .00 |
|  | P × R | 1 | 248 | 0.25 | .615 | .00 |
|  | E × R | 1 | 248 | 0.06 | .814 | .00 |
|  | S × R | 1 | 248 | 14.71*** | .000 | .06 |
|  | P × E × S | 1 | 248 | 0.06 | .812 | .00 |
|  | P × E × R | 1 | 248 | 1.23 | .268 | .00 |
|  | P × S × R | 1 | 248 | 11.37** | .001 | .04 |
|  | E × S × R | 1 | 248 | 0.65 | .421 | .00 |
|  | P × E × S × R | 1 | 248 | 0.01 | .920 | .00 |
|  | *Note.* *df*= degrees of freedom. **p*<.05. ***p*<.01. ****p*<.001. | | | | | |

**Supplementary Analysis III: Did participants not believe the cover story?**

Many participants in the human partner condition reported that they interacted with computer in the post-experimental questionnaire. We thus analysed whether retrieval effects were reduced or absent for these individuals, which would indicate that participants did not show effects because they second-guessed the manipulation during the study or did not believe the cover story right from the start. We analysed whether retrieval effects differed among participants in the human partner condition, depending on which interaction partner was reported in the post-experimental questionnaire. Only participants of the human partner condition of both studies entered into the analysis who reported that they interacted with a human (n= 84) or computer (n=26). A 2 (Experiment) × 2 (Reported interaction partner) factorial ANOVA on retrieval effect scores yielded no significant results (reported interaction partner: *F*[1,106]=1.65, *p*=.202, , ηp²=.02; experiment: *F*[1,106]=0.36, *p*=.550, , ηp²<.01, interaction: *F*[1,106]=0.47, *p*=.496, ηp²<.01, see also Figure S1). Based on these null findings, it can be ruled out that participants second-guessed the nature of the study. If that were the case, retrieval effects should have been reduced or even absent for participants who reported to have interacted with a computer instead of a human partner; instead, the descriptive data pattern showed the reversed trend (albeit nonsignificant). It is thus more likely that the reported interaction partner responses reflect demand effects.

*
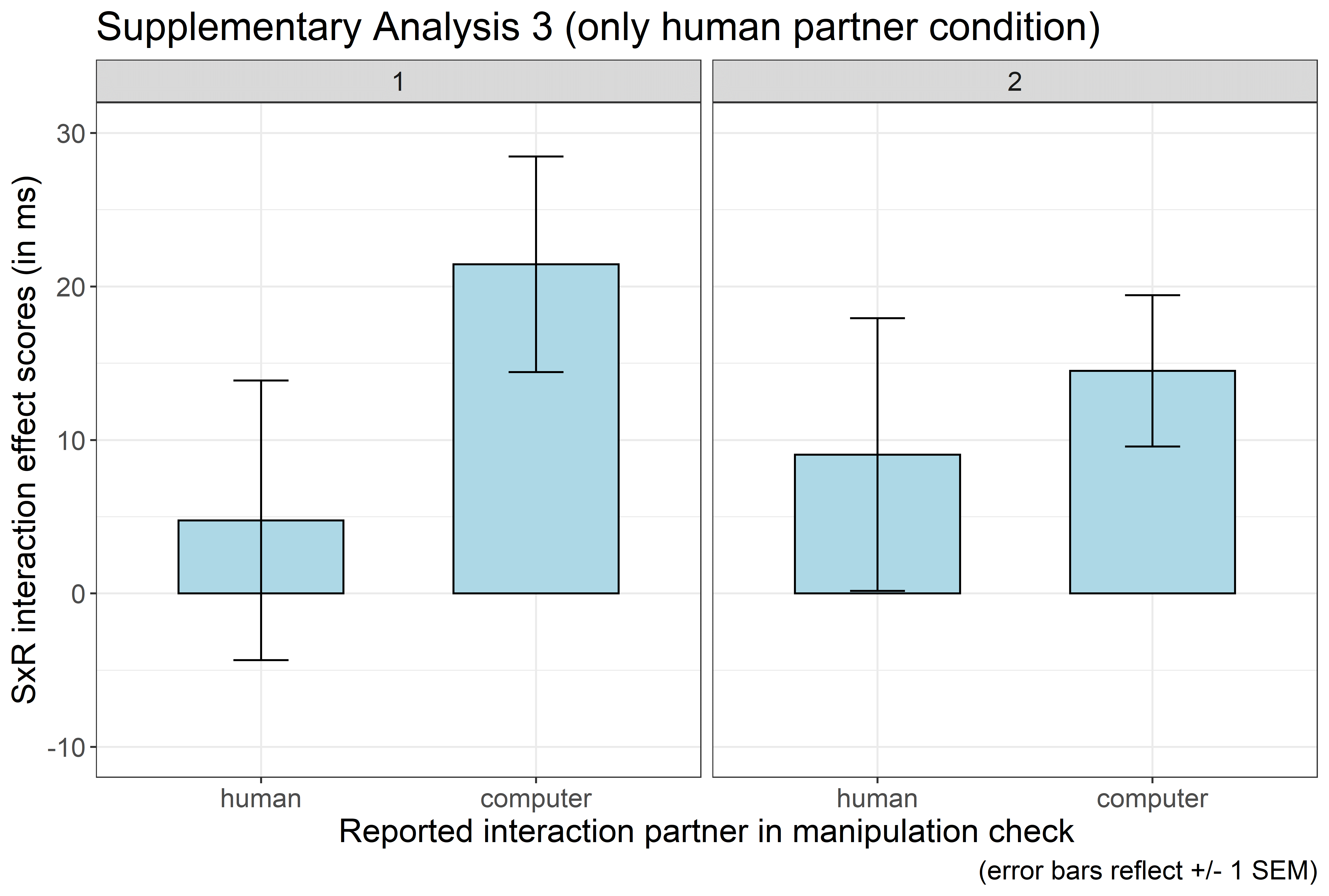
Figure S1*

*Figure S1. S×R interaction effect scores (in ms; see Table 2 in main text for effect computation) for participants of the human partner condition as a function of Experiment and Reported Interaction Partner in the post-experimental questionnaire. Positive values reflect effects that are in line with retrieval of observationally acquired stimulus-response episodes (i.e., benefits due to SR retrieval on compatible probe trials and costs due to SR retrieval on incompatible probe trials, see also captions of Figure 2 in the main text).*

**Supplementary Analysis IV: Did memory tests enhance the distinctiveness of subsequent prime displays?**

For participants in the human partner condition, 25% of all memory test displays were followed by a waiting display to yield the impression that the alleged interaction partner did not yet finish the test. Unexpectedly, this might have resulted in a systematic advantage for subsequent prime-probe sequences, as these were now more distinct and easier to separate from the remaining prime-probe sequences. As waiting displays appeared only in the human partner condition, this memory advantage poses an alternative explanation for stronger retrieval effects in this condition. To systematically address whether there is merit to this explanation, we coded for each prime-probe sequence whether the preceding sequence contained a memory test. Then, we analysed interaction effect scores as a function of partner condition and previous sequence type in a 2 (condition: human partner vs. computer) × 2 (previous sequence: memory test present vs. absent) mixed factors ANOVA on the joint data set (one participant in Exp. 2 had to be excluded due to empty cells). The analysis yielded a significant intercept, *F*(1,248)=4.75, *p*=.030, ηp²=.02 (meaning that effect scores differed significantly from zero averaged across all other factors) that was qualified by a main effect of condition, *F*(1,248)=4.12, *p*=.044, ηp²=.02 which reflected the pattern reported in the main analyses, see also Figure S2. Neither previous sequence, nor its interaction with condition was significant, *F*(1,248)=1.15, *p*=.284, ηp²<.01 and *F*(1,248)=0.25, *p*=.616, ηp²<.01, respectively.

For both experiments, these results clearly argue against the alternative explanation that occasional waiting displays in the human partner condition rendered the subsequent prime-probe sequence as more distinct and thus selectively facilitated memory retrieval in this condition.

*
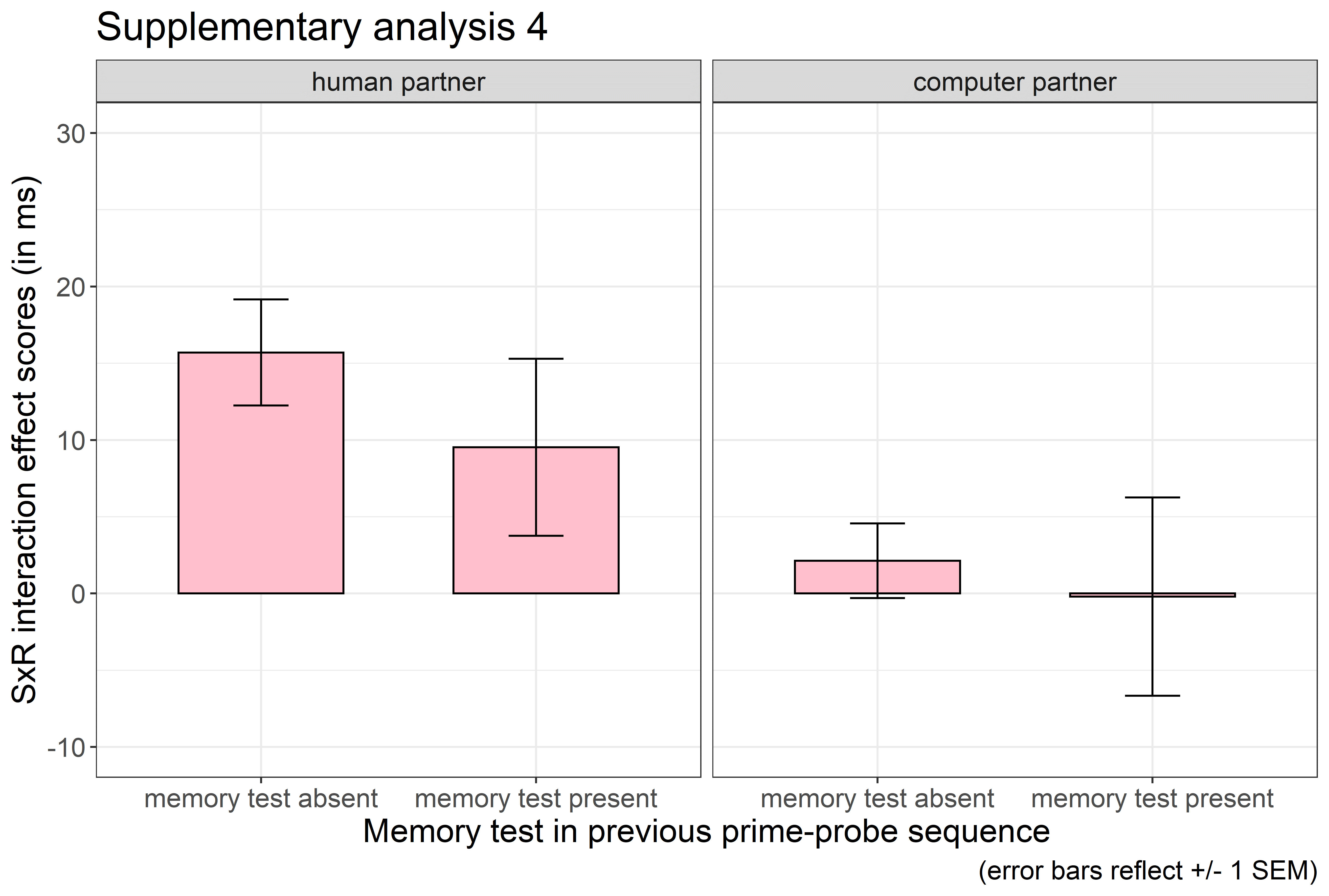
Figure S2*

*Figure S2. S×R interaction effect scores (in ms; see Table 2 in main text for effect computation). Positive values reflect effects that are in line with retrieval of observationally acquired stimulus-response episodes (i.e., benefits due to SR retrieval on compatible probe trials and costs due to SR retrieval on incompatible probe trials). As can be seen, retrieval effect scores were always present for participants who believed to be interacting with a human partner. Importantly, these effects did not depend on whether a memory test occurred in the preceding prime-probe sequence or not. For participants who interacted with the computer, retrieval effects were absent.*
